# Rathke’s cleft cyst discovered with ruptured anterior communicating artery aneurysm: a case report

**DOI:** 10.1186/s13256-023-04115-5

**Published:** 2023-09-12

**Authors:** Nipun Lakshitha de Silva, Hemantha Gunathilaka, Saman Wadanamby, Manilka Sumanatilleke, Noel Somasundaram

**Affiliations:** 1 Department of Clinical Sciences, Faculty of Medicine, General Sir John Kotelawala Defence University, Ratmalana, Sri Lanka; 2 Diabetes and Endocrine Unit, National Hospital of Sri Lanka, Colombo 10, Sri Lanka; 3 Neurosurgical Unit, National Hospital of Sri Lanka, Colombo 10, Sri Lanka

**Keywords:** Rathke’s cleft cyst, Anterior communicating artery aneurysm, Subarachnoid hemorrhage

## Abstract

**Introduction:**

Rathke’s cleft cysts are thought to have a benign clinical outcome apart from associated hypopituitarism and visual defects. Synchronous central nervous system lesions, including pituitary adenoma and intracerebral aneurysms, are rarely reported. Diagnosis of Rathke’s cleft cyst after presenting with a subarachnoid hemorrhage due to a ruptured arterial aneurysm is reported only once before.

**Case presentation:**

A 33-year-old Sri Lankan female presented with a subarachnoid hemorrhage due to a ruptured anterior communication artery aneurysm. She underwent pterional craniotomy and aneurysm clipping. She was found to have partial cranial diabetes insipidus and hypogonadotropic hypogonadism. She had a cystic lesion occupying enlarged sella turcica with characteristics of a Rathke’s cleft cyst. Subsequently, she underwent trans-sphenoidal excision of the sellar lesion. Histology confirmed the diagnosis of Rathke’s cleft cyst.

**Conclusions:**

Rare co-occurrence of a Rathke’s cleft cyst and an anterior communicating artery aneurysm would have been missed if subtle manifestations atypical for subarachnoid hemorrhage were not further pursued. This could have led to progressive visual deterioration and hypopituitarism.

## Background

Rathke’s cleft cyst (RCC) is a benign non-neoplastic cystic lesion occurring in the sellar or suprasellar region from the remnant of Rathke’s pouch [1]. Most are small intrasellar lesions detected incidentally and are clinically silent. However, larger lesions and those with suprasellar extension can present with headache, hypopituitarism, and visual impairment [1, 2]. Pathology related to RCC is limited to sellar and suprasellar regions [3]. Once the cyst is surgically removed, follow-up is focused on detecting recurrences and managing endocrine effects. There had been no concern about possible synchronous intracranial lesions in these patients.

Most of the large series reporting patients with RCC do not report co-occurrence of any intracranial pathologies [1, 4–6]. There are only several reports of synchronous lesions, including pituitary adenoma and hypothalamic hamartoma [2, 7]. The presence of cerebral aneurysms in patients with RCC seems to be even rare and reported in few case reports only.

We report a young female who initially presented with subarachnoid hemorrhage (SAH) due to a ruptured anterior communicating artery (ACOM) aneurysm unveiling the diagnosis of RCC.

## Case presentation

A 33-year-old Sri Lankan woman presented with sudden-onset severe headache, neck pain, vomiting, and transient loss of consciousness. On presentation, she was in pain, alert, and oriented. There was neck stiffness. There were no neurological deficits including normal vision. Pulse rate was 80 beats per minute, and blood pressure was 130/70 mmHg. The rest of the examination was normal.

**Fig. 1:**
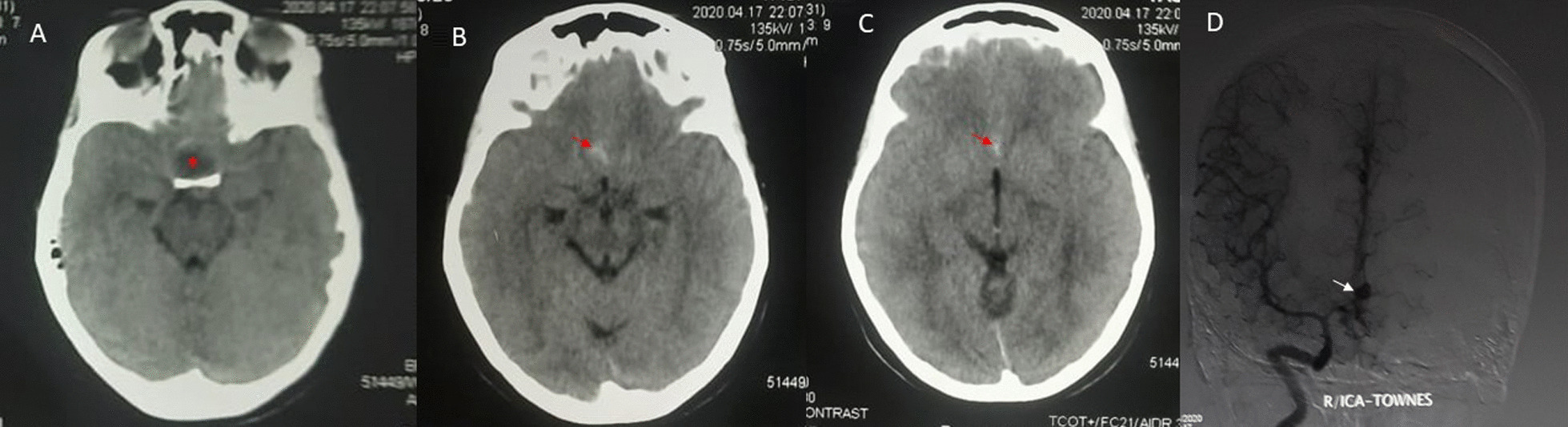
Non-contrast computed tomography of the head performed on initial presentation showing high-density signals in the basal cisterns suggestive of acute subarachnoid hemorrhage (**B**, **C**, red arrow). There is a nonspecific hypodensity in the sellar region (**A**, red asterisk). Cerebral digital subtraction angiogram performed during the initial presentation shows a bi-lobed secular aneurysm of the anterior communicating artery with the aneurysm sac measuring 3.7 × 4 mm anteriorly and laterally (**D**, white arrow)

Urgent non-contrast computed tomography (CT) of the head showed high-density areas in the basal cisterns suggestive of acute subarachnoid hemorrhage (SAH). There was nonspecific hypodensity in the sellar area (Fig. 1A–C). Digital subtraction angiogram (DSA) showed a bi-lobed saccular ACOM aneurysm (Fig. 1D). The aneurysm opacified predominantly during right internal carotid artery (ICA) injection. She was in grade 1 of World Federation of Neurological Surgeons (WFNS) classification scale for SAH. She underwent right pterional craniotomy and ACOM aneurysm clipping 9 days from the onset of symptoms. A routine aneurysm clipping surgery was performed without any intraoperative adverse events. Any mass suspicious for a sellar lesion was not found in the vicinity during surgery.

Pre- and postoperative periods were complicated with partial cranial diabetes insipidus. After recovery from the surgery, on further questioning, it was revealed that she has had polyuria, polydipsia, nocturia, and secondary amenorrhea for 6 months before the presentation. She has not sought any medical advice for the symptoms. She had no other symptoms or co-morbidities. She has not had any intracranial lesions in the past.

She was started on desmopressin, which produced improvement of symptoms. Pituitary hormone profile and pituitary magnetic resonance imaging (MRI) were performed after recovery from the acute event. Low gonadotropins for an amenorrheic female with low estradiol were noted 6 weeks after the surgery. Partial cranial diabetes insipidus persisted. Biochemical investigation results are summarized in Table 1. There was bitemporal upper quadrantanopia on visual field testing.

**Table 1:** Summary of biochemical test during the presentation

Investigation	Result	Reference range
Serum creatinine (mg/dL)	0.69	0.5–1.1
Sodium (mmol/L)	143	135–145
Potassium (mmol/L)	3.8	3.5–5.1
Fasting plasma glucose (mg/dL)	89	< 100
Thyroid-stimulating hormone (mIU/L)	0.564	0.5–4.7
Free T4 (ng/dL)	1.1	0.89–1.76
9 am cortisol (nmol/L)	329.97	118–618
Prolactin (mIU/L)	560	59–619
Follicle-stimulating hormone (IU/L)	5.86	
Luteinizing hormone (IU/L)	4.64	
Serum estradiol (pmol/L)	131	
Extended water deprivation test		
Serum osmolality before desmopressin (mOsm/L)	297	
Serum sodium before desmopressin (mmol/L)	146	
Urine osmolality before desmopressin (mOsm/L)	358	
Urine osmolality after desmopressin (mOsm/L)	464	
β-hCG (mIU/L)	< 2	< 5
α-Fetoprotein (IU/mL)	4.4	< 8.1

**Fig. 2:**
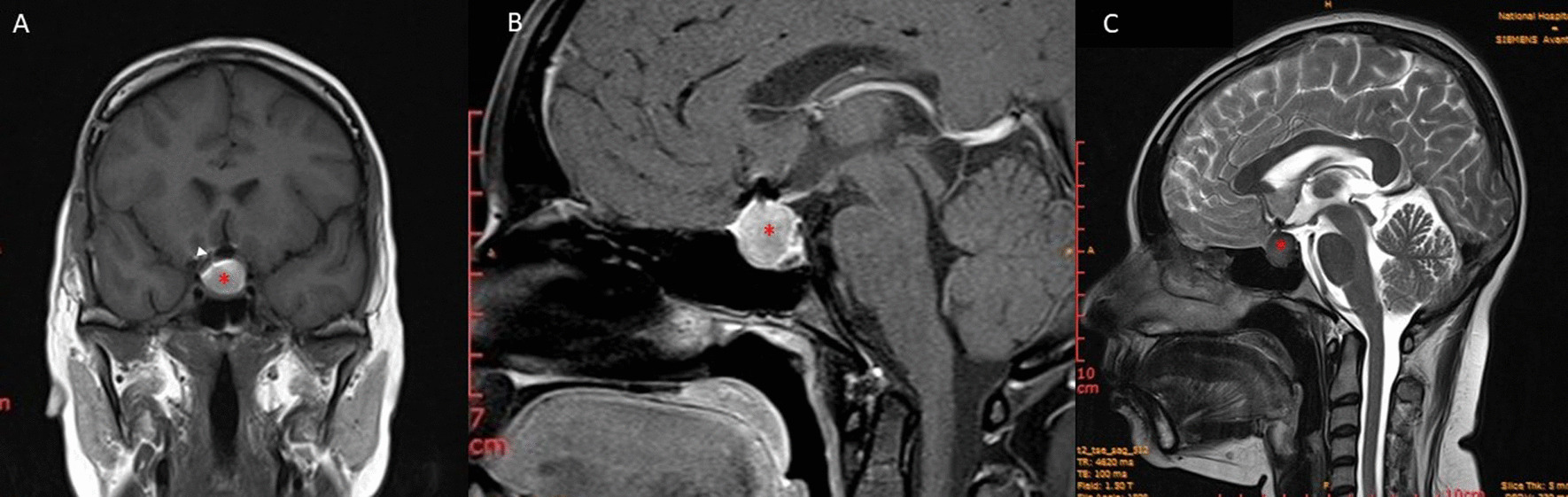
Coronal (**A**) and sagittal (**B**) T1 post-contrast and T2 sagittal (**C**) sections of the magnetic resonance imaging of the pituitary region showing 1.5 cm × 1.7 cm × 1.7 cm cystic mass in the enlarged sella turcica (asterisk). The mass is T1 hyperintense and T2 hypointense. It has suprasellar extension causing optic chiasmal compression (white arrowhead). There is no contrast enhancement

MRI-pituitary showed a 1.5 cm × 1.7 cm × 1.7 cm lesion occupying enlarged sella turcica with suprasellar extension and optic chiasmal compression. This lesion had high signal intensity on T1 and low signal intensity on the T2 phases of MRI. There was no contrast enhancement. (Fig. 2). A recurring or residual aneurysm was excluded with a CT angiogram.

She underwent trans-sphenoidal excision of the sellar lesion. The dural defect was repaired with a fascia lata graft harvested from the right thigh in combination with an artificial dural substitute. She was commenced on hydrocortisone replacement following surgery. Morning cortisol was 341 nmol/L, and free T4 was 0.603 ng/dL (0.93–1.7). Hydrocortisone, levothyroxine, and desmopressin were continued. The recovery was uneventful.

Histology showed cyst wall focally lined by stratified columnar epithelium. Wall showed sheets of foamy cells with multinucleated giant cells and pigment-laden foamy cells. Normal pituitary tissue was seen. There was no evidence of a pituitary tumor. Features were in favor of a Rathke’s cleft cyst.

Three-month postoperative MRI did not show any residual masses. She remained amenorrheic with low gonadotropins. She was started on combined oral contraceptive pills. At 1 year follow-up, she was clinically well while on levothyroxine and oral contraceptive pills. Hydrocortisone and desmopressin were omitted due to clinical and biochemical evidence of resolution of adrenocorticotrophic hormone and vasopressin, respectively. She is stable and satisfied with her current clinical status with a Global Physical Health Raw Score of 18/20 (90%) and Global Mental Health Raw Score of 16/20 (80%) according to the patient-reported outcomes measurement information system (PROMIS) scale [8].

## Discussion

Our patient presented to the healthcare due to SAH, and the diagnosis of RCC was made subsequently even after the surgery for the ACOM aneurysm. Adipsic diabetes insipidus has been reported in patients following SAH [9] and clipping of the ACOM aneurysms [10]. However, diabetes insipidus is unusual in patients with ACOM aneurysms that have not ruptured. The presence of secondary amenorrhea and diabetes insipidus before the SAH prompted us to pursue an alternative diagnosis. Pituitary hormone evaluation, visual field assessment, and pituitary imaging were performed due to this concern. Otherwise, missed RCC could have led to progressive visual impairment and hypopituitarism. Knowledge of these rare associations would have helped to be more vigilant during the assessment. This could have enabled surgical intervention during a single attempt.

A report from Japan describes the presentation of a 44-year-old man with a sudden-onset headache due to subarachnoid hemorrhage. Evaluation has revealed a ruptured aneurysm in the A1 portion of the left anterior cerebral artery and an RCC [11]. This is the only available case report of a patient with previously undiagnosed RCC presenting with SAH. He underwent neck clipping of the aneurysm and opening of the RCC during single surgical procedure in contrast to our patient.

There are few case reports in the literature reporting RCC and cerebral aneurysms occurring in the same patient, though none of the large case series in international literature describes this association. In a Japanese case series, five patients with intracranial aneurysms and RCC were mentioned according to the information in the abstract [12]. However, detailed review could not be performed since full-text article was not available.

In most of the reported cases, aneurysm was detected subsequently when the patient presented with features of RCC. A 57-year-old female who underwent trans-sphenoidal surgery for an RCC developed SAH 3 days later from a very small aneurysm in the superior hypophyseal artery [13]. Authors speculate that preoperatively undiagnosed small aneurysms adherent to the RCC might have been stretched due to the deflation of the cyst. A 66-year-old female presenting with visual symptoms was found to have a large RCC and bilateral ICA aneurysms in C2 portions [14]. In a single operative approach through bi-frontal craniotomy, the RCC was removed, and bilateral aneurysms were clipped. A 60-year-old female has presented with complete temporal hemianopia of the left eye and incomplete temporal hemianopia of the right eye. She was found to have an RCC in the intrasellar and suprasellar regions with compression of the optic nerves and optic chiasm. The aneurysmal dome of the ACOM aneurysm was stuck in the central region of the chiasm [15].

One case report describes a patient with an RCC and intracranial aneurysms diagnosed after presenting with a stroke [16]. This patient has had two aneurysms: the first one at the beginning of the C6 segment of the left internal carotid artery (ICA) projecting inferomedially and indenting the RCC, and the second one at the junction of the left A1 and A2 segments. The first aneurysm was removed together with the RCC through an endoscopic trans-nasal approach.

Whether the association of an RCC and an ACOM aneurysm purely represents random co-occurrence or any pathophysiological association remains elusive. A similar association was noted in patients with pituitary adenoma for the past several decades. There is possible evidence suggesting a higher prevalence of aneurysms among patients with pituitary adenoma than that of the general population [17]. This may be due to associations such as the direct mechanical effect of the tumor on the vasculature or direct infiltration of the tumor. The pituitary gland derives its blood supply from superior and inferior hypophyseal arteries, which are direct branches of the cavernous segment of the internal carotid artery, but the anterior segment of the hypothalamus gains its blood supply from branches of the anterior cerebral and anterior communicating arteries [18]. However, the blood vessels and pituitary arise from two separate germ layers from an embryological point. The pituitary and RCC develop from an outpouching of ectoderm from Rathke’s pouch, whereas blood vessels derive from internal carotid arteries arising from the mesoderm. Therefore, it is difficult to explain this through possible developmental malformation.

There were few limitations experienced during the management of this patient due to resource limitations and work demand. Ideally, aneurysm clipping could have been performed as early as possible. However, patient initially presented to a center without neurosurgical facilities, and after the transfer, arranging DSA and surgical intervention required substantial time. Hydration and nimodipine were continued to overcome vasospasm. There was some nonspecific hypodensity in the sellar region, though this did not receive much attention during the acute phase where the focus was on SAH. Routine radiology reporting of all non-contrast CT scans of the head is not available due to limited specialist capacity.

## Conclusions

While co-occurrence of RCC and aneurysms is rare, this combination initially presenting with SAH is exceptional. Thorough attention to subtle clinical clues will prevent delayed diagnosis and risk of progressive hormonal and visual deterioration.

## Data Availability

Data sharing is not applicable to this article as no datasets were generated or analyzed during the current study.

## Abbreviations

ACOM: Anterior communicating
ICA: Internal carotid artery
MRI: Magnetic resonance imaging
RCC: Rathke’s cleft cyst
SAH: Subarachnoid hemorrhage
TSH: Thyroid-stimulating hormone
